# Supplementary material for: Biophysical Parameters of Plasma-Derived Extracellular Vesicles as Potential Biomarkers of Bone Disturbances in Breast Cancer Patients Receiving an Individualized Nutrition Intervention
Source: Nutrients. 2023 Apr 19;15(8):1963. doi: 10.3390/nu15081963 (PMC10141602; doi:10.3390/nu15081963)
Supplement: Supplementary file 1 [file nutrients-15-01963-s001.zip › nutrients-2311622-supplementary.pdf]

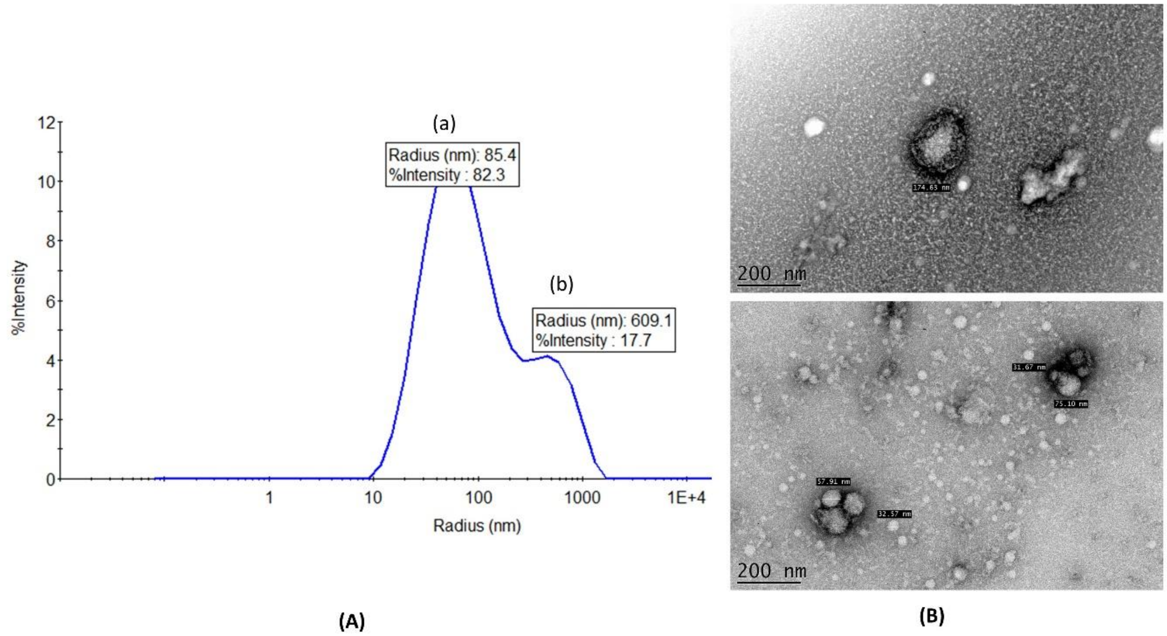

Figure S1. Characterization of extracellular vesicles isolated from plasma of breast cancer patients. (A) Example of one of the regularization graphs of the particles isolated from the plasma of one of the participants measured by dynamic light scattering. We can observe two peaks, (a) indicates the particles identified as small extracellular vesicles population and (b) represents the particles identified as large extracellular vesicles population. (B) Particles isolated from plasma of the participants analyzed by transmission electron microscopy.
